# How the Innate Immune System of the Blood Contributes to Systemic Pathology in COVID-19-Induced ARDS and Provides Potential Targets for Treatment

**DOI:** 10.3389/fimmu.2022.840137

**Published:** 2022-03-08

**Authors:** Bo Nilsson, Barbro Persson, Oskar Eriksson, Karin Fromell, Michael Hultström, Robert Frithiof, Miklos Lipcsey, Markus Huber-Lang, Kristina N. Ekdahl

**Affiliations:** ^1^ Department of Immunology Genetics and Pathology, Uppsala University, Uppsala, Sweden; ^2^ Department of Surgical Sciences, Anesthesiology and Intensive Care, Uppsala University, Uppsala, Sweden; ^3^ Unit for Integrative Physiology, Department of Medical Cell Biology, Uppsala University, Uppsala, Sweden; ^4^ Hedenstierna Laboratory, Anesthesiology and Intensive Care, Department of Surgical Sciences, Uppsala University, Uppsala, Sweden; ^5^ Institute for Clinical and Experimental Trauma-Immunology, University Hospital of Ulm, Ulm, Germany; ^6^ Linnaeus Centre for Biomaterials Chemistry, Linnaeus University, Kalmar, Sweden

**Keywords:** cascade system, leukocytes, platelets, plasma proteins, COVID-19

## Abstract

Most SARS-CoV-2 infected patients experience influenza-like symptoms of low or moderate severity. But, already in 2020 early during the pandemic it became obvious that many patients had a high incidence of thrombotic complications, which prompted treatment with high doses of low-molecular-weight heparin (LMWH; typically 150-300IU/kg) to prevent thrombosis. In some patients, the disease aggravated after approximately 10 days and turned into a full-blown acute respiratory distress syndrome (ARDS)-like pulmonary inflammation with endothelialitis, thrombosis and vascular angiogenesis, which often lead to intensive care treatment with ventilator support. This stage of the disease is characterized by dysregulation of cytokines and chemokines, in particular with high IL-6 levels, and also by reduced oxygen saturation, high risk of thrombosis, and signs of severe pulmonary damage with ground glass opacities. The direct link between SARS-CoV-2 and the COVID-19-associated lung injury is not clear. Indirect evidence speaks in favor of a thromboinflammatory reaction, which may be initiated by the virus itself and by infected damaged and/or apoptotic cells. We and others have demonstrated that life-threatening COVID-19 ARDS is associated with a strong activation of the intravascular innate immune system (IIIS). In support of this notion is that activation of the complement and kallikrein/kinin (KK) systems predict survival, the necessity for usage of mechanical ventilation, acute kidney injury and, in the case of MBL, also coagulation system activation with thromboembolism. The general properties of the IIIS can easily be translated into mechanisms of COVID-19 pathophysiology. The prognostic value of complement and KKsystem biomarkers demonstrate that pharmaceuticals, which are licensed or have passed the phase I trial stage are promising candidate drugs for treatment of COVID-19. Examples of such compounds include complement inhibitors AMY-101 and eculizumab (targeting C3 and C5, respectively) as well as kallikrein inhibitors ecallantide and lanadelumab and the bradykinin receptor (BKR) 2 antagonist icatibant. In this conceptual review we discuss the activation, crosstalk and the therapeutic options that are available for regulation of the IIIS.

## Introduction

COVID-19 has been shown to have a multifaceted effect on the immune system. In a recently published article, we reported that the innate immune system of the blood, here designated the intravascular innate immune system (IIIS), is strongly activated in severe COVID-19 with ARDS (1), which is the major explanation for the serious course of the disease. In this article we review the IIIS and how its physiological function contributes to tissue injury and to the proinflammatory state during severe COVID-19. Finally, we review potential treatment targets within the IIIS with existing drugs that could be expected to modulate the disease course in COVID-19.

## The Intravascular Innate Immune System (IIIS)

The blood contains a large number of plasma proteins and cells that constitute our innate barrier both in terms of recognition and elimination of microorganisms. Here, we define the IIIS and focus on the network of proteins and cells that forms the innate immune system in blood leading to thromboinflammation (2). The IIIS consists of the cascade system of the blood: the complement system, the coagulation system, the kallikrein/kinin (KK; or contact system), and the fibrinolytic system. Also, individual proteins related to the cascade systems such as collectins, pentraxins, etc. belong to the IIIS, as do blood cells, e.g., granulocytes, monocytes, platelets and endothelial cells. These proteins are also available in the mucous membranes of the body, especially during inflammation either by passive diffusion or by active synthesis in the lining epithelial cells, while cells, such as granulocytes and monocytes, are recruited by chemotaxis mediated by the anaphylatoxins (C3a, C5a), by bradykinin (BK) and by chemokines.

Over the years, IIIS research has been very separated, not only in the laboratory but also in the collaboration between researchers and in the literature. For obvious reasons, human blood samples have been used for the studies in the various disciplines of the IIIS, and since the blood needs to be anticoagulated to be separated into a fluid phase and blood cells, different anticoagulants have been used. Divalent cations are necessary for IIIS function: the coagulation cascade requires Ca^2+^ and the complement cascade both Ca^2+^ and Mg^2+^ ions, which affects the usage of anticoagulant for the different cascade system. As a consequence, EDTA (chelating both Ca^2+^ and Mg^2+^) is used for assessment of complement activation fragments/products in plasma, while the corresponding anticoagulant for the coagulation, contact and fibrinolytic systems is citrate (chelating Ca^2+^ ions, but not Mg^2+^). Studies of the complement system function is performed using serum (i.e., the remaining fluid-phase after blood is coagulated), whereas for the coagulation, KK, and fibrinolysis systems, citrate reconstituted with Ca^2+^ ions is the plasma preparation of choice. These pre-analytic procedures have totally separated the research disciplines. Further aggravating the problem, cell studies have in many cases been performed on heparin blood, where activation of the coagulation and complement systems are considerably dampened by the high concentrations of heparin. Consequently, there is no generally used holistic assay format available for simultaneous assessment of all IIIS functions. However, for specialized *in vitro* studies heparin-coated tubes or tubing can be used, leaving the blood fresh and unaffected (3). In this conceptual review the intension is to consider these components of the blood as an intact network that gives rise to an integrated innate immune response in late-stage COVID-19. The findings by us and others support this approach.

## Organization of the IIIS

**Figure 1:**
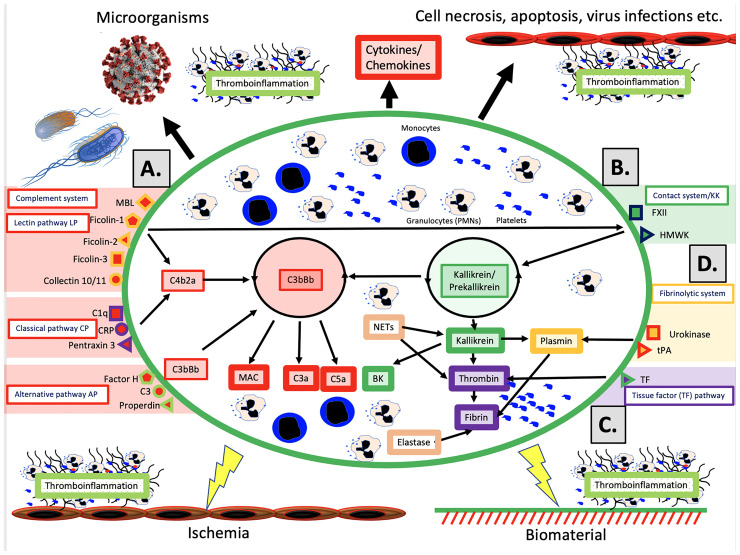
Physiological and pathological conditions and treatments involving IIIS activation. The IIIS consists of the cascade systems of the blood: the complement system **(A)**, the contact or kallikrein/kinin system (KKS) **(B)**, the tissue factor (TF) pathway of the coagulation system **(C)**, and the fibrinolytic system **(D)**. Activation of IIIS occur in response to physiological stimuli such as microorganisms or necrotic, apoptotic or virus infected cells (top in figure), which leads to thromboinflammation. But also during pathological or therapeutic conditions such as ischemia during transplantation, or treatment with biomaterials e.g. stents, intravascular devices, and extracorporeal treatment (bottom in figure) a similar reaction occur. **(A)** The complement system has three activation pathways, which are triggered by different recognition molecules. The classical pathway (CP) is initiated by C1q, which binds to antigen bound IgG and IgM, but also to negatively charged surfaces and target-bound pentraxins e.g. CRP and pentraxin 3. The lectin pathway (LP) is activated by a number of carbohydrate binding proteins (lectins) such as mannan binding lectin (MBL), Ficolin-1, -2, -3 and Collectin 10/11 (4). The alternative pathway (AP) is be activated/regulated by surface-specific binding of factor H, C3 or properdin to a target, and has its main role as an amplifier. Complement activation leads to assembly of two enzyme complexes C4bC2a and C3bBb, which cleave C3 into the anaphylatoxin C3a and surface bound C3b (opsonization) and cleaves C5, which initiates the formation of the membrane attack complex (MAC) and the more potent anaphylatoxin C5a. **(B)** The primary function of the kallikrein/kinin and coagulation systems is in hemostasis but both systems are also engaged in inflammation. The recognition molecule in the contact/KK system is factor XII (FXII), which is activated, e.g., by negatively charged molecules such as LPS, glycosaminoglycans or extracellular matrix molecules exposed to the blood stream. The KK system also initiates an amplification loop, which involves prekallikrein that cleaves high molecular-weight kininogen (HMWK) leading to the generation of the proinflammatory mediator bradykinin (BK). **(C)** The main physiological trigger of coagulation, tissue factor (TF), is exposed in a functionally active form only after damage to vessels and activation of blood cells including platelets. It thereby initiates the extrinsic part of the coagulation cascade, which leads to formation of high amounts of thrombin. **(D)** The fibrinolytic system is initiated when urokinase or tissue plasminogen activator (tPA) activate plasminogen to plasmin, which degrades a formed fibrin network to soluble fibrin fragments. Please see the text for information on the roles of monocytes, PMNs and platelets.

The schematic structure of the IIIS is described in **Figure 1**, which focuses on the interaction of the cascade systems of the blood, i.e., the complement, coagulation, KK and fibrinolytic systems. The reason for highlighting the cascade systems is that they contain most of the recognition molecules that specifically target DAMPs and PAMPs and trigger activation of the entire IIIS. In the figure, they are marked as components of the activation pathways that they initiate. The complement system has three defined activation pathways: the classical (CP), the lectin pathways (LP) and the alternative (AP) that are triggered by different stimuli (5).

- The CP is initiated by C1q, which binds to negatively charged surfaces, to IgG and IgM in immune complexes, and to target-bound pentraxins such as CRP and pentraxin 3.
- The LP is activated by a number of lectins (i.e., carbohydrate-binding proteins) such as MBL, Ficolin-1, -2, and -3, and by the Collectins 10/11 (4).
- The AP functions primarily as an amplification loop but can be specifically regulated by properdin in concert with C3 and by factor H as an important recognition molecule controlling the AP convertase.

*In vivo*, the coagulation system is mainly activated by the extrinsic pathway elicited by tissue factor (TF), which is exposed in the vessel wall after endothelial cell damage. TF is also expressed by multiple cells in response to inflammatory signals (6) and when cell-bound TF is exposed to blood it leads to a strong coagulation activation. Active TF can also be expressed by activated monocytes in response to, e.g., C5a and on the endothelium during inflammation. The KK system is an alternative route for coagulation activation, initiated on contact between blood and negatively charged foreign surfaces. Activation of the contact system is the reason why blood collected without an anticoagulant, coagulates in a test tube. KK is also regulating the vascular permeability on endothelial cells (7).

Factor XII (FXII) has a dual role in that it is the starting point of both the intrinsic pathway activation of the coagulation system and KK system. FXII probably has a limited role in physiological hemostasis, which is illustrated by the fact that FXII deficiency (Hageman disease) does not lead to an increased tendency for bleeding (8). In contrast, inhibition of FXII is considered as a new possible strategy as an antithrombotic drug with minor risk for bleeding (9). The KK system consists of FXII, prekallikrein and high molecular weight kininogen (HMWK) (8). FXIIa cleaves prekallikrein generating kallikrein. Kallikrein can cleave FXII and prekallikrein thereby providing a positive feedback loop. The KK system elicits inflammation *via* kallikrein, which cleaves HMWK generating BK.

The fibrinolytic system is initiated by urokinase, tissue plasminogen activator (tPA) and FXIIa by activating plasminogen to plasmin (10). Activated plasmin in turn breaks down the fibrin network formed in the final stages of the coagulation reaction, and thus acts as a physiological restriction mechanism that limits the propagation of the clot (10).

## The Function of the IIIS

Significant cross-activation can take place directly or indirectly *via* leukocytes and platelets, which means that activation of one of the cascade systems can spread to the entire IIIS. The physiological end result of IIIS activation is thromboinflammation, which stops bleeding by sealing blood vessel leakage through fibrin formation and platelet aggregation, and supports the clearance of damaged cells by attracting leukocytes that remove the damaged tissue. The IIIS is the start of wound healing after an injury, where the “waste management function”, i.e., removal of foreign substances, particles and apoptotic or necrotic cells is an important task. This process is combined with the release of growth factors from, e.g., activated platelets, which ultimately leads to tissue healing and scar formation (11).

In a similar way, the IIIS reacts to infections caused by different types of microorganisms. In these reactions, the IIIS helps to kill and remove the microorganisms or the infected cells, after which the tissue is cleansed and healed as a result of IIIS functions. Sometimes, however, the reaction can shoot over the target and become too strong, which leads to severe inflammation and tissue damage. The end result is an excessive and pathological thromboinflammation with activation of all IIIS components such as in sepsis (**Figure 1**).

## COVID-19 and IIIS Activation Leading to Thrombinflammation

The COVID-19 pandemic, which is caused by the corona virus SARS-CoV-2 was initially described in Wuhan, China at the end of 2019 and has since had an immense effect on human society worldwide. Most SARS-CoV-2 infected patients experience influenza-like symptoms of low or moderate severity that are characterized by sore throat, fever, a dry cough, intestinal problems, and a loss of taste and smell. Early in the pandemic, it was reported that most patients suffered from an acute-phase reaction, with high levels of certain plasma proteins, e.g., fibrinogen, C3, and ferritin that were sometimes multiple times higher than normal (12). It also became clear that there was an increased risk of severe thromboembolism (13, 14). In some patients, the disease aggravated after approximately 9 to 10 days and turned into a full-blown ARDS-like pulmonary inflammation with endothelialitis, thrombosis and vascular angiogenesis, which often required intensive care treatment with ventilator support (15). The lung injury in COVID-19 resembles the pulmonary complications that have been observed in earlier SARS-1 and MERS epidemics (16). In the beginning of the COVID pandemic, up to 30% of the intensive care unit (ICU) patients died due to pulmonary and other organ dysfunctions. Those patients who reached the late-stage syndrome developed a severe pulmonary inflammation with general cyto-/chemokine expression (17), reduced oxygen saturation, increased risk of pulmonary thrombosis, and signs of severe pulmonary damage revealed by CT, particularly in the periphery of the lungs (18, 19).

In 2020, we studied the first 65 patients with intensive care-requiring COVID-19 admitted to the ICU of the university hospital in Uppsala, Sweden (1). These patients were admitted to the ICU before the therapeutic procedures now used, such as corticosteroids, were introduced. These patients were comprehensively investigated and were found to have a pronounced activation of the IIIS, with activation of all cascade systems and especially the KK system with prekallikrein, FXII and HMWK consumption (i.e. activated) and kallikrein/C1-INH complex formation (1).

## Thromboinflammatory Changes in Various Tissues of COVID-19 Patients

**Figure 2:**
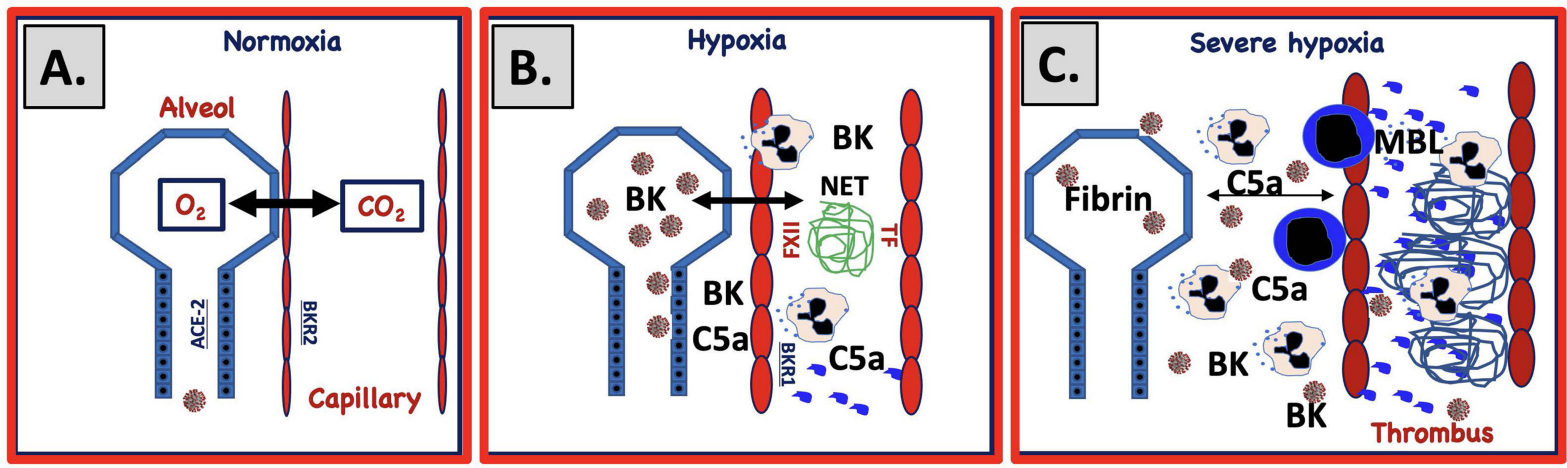
Proposed mechanism for IIIS involvement in SARS-CoV-2-induced ARDS. **(A)** Under normal circumstances gas is exchanged over a narrow gap between alveolar epithelial cells (blue) and capillary endothelial cells (red). SARS-CoV-2 infects the alveolar/bronchial epithelial cells *via* ACE-2. Bronchial epithelial cells express ACE-2 and endothelial cells bradykinin receptor 2 (BKR2). Infection of alveolar epithelial cells activates the complement and KK systems and generates C5a and bradykinin (BK). **(B)** C5a and BK activate endothelial cells and elicit increased vascular permeability, which widens the gap between the cell linings. Activated endothelial cells also trigger complement and KK systems activation that upregulates BKR1, further increasing vascular permeability, damaging cells and inducing necrosis and apoptosis. BK and C5a elicit chemotaxis of PMNs that release neutrophil extracellular traps (NETs, depicted as a blue mesh). **(C)** Activated endothelial cells (TF) and NETs (TF and FXIIa) trigger coagulation activation and thrombus formation and further amplifie KK and complement activation. Plasma proteins leak into the alveolae causing fibrin precipitation. Invasion of PMNs and monocytes further increases the gap between the cell linings, ultimately leading to a collapsed exchange of gases over the epithelial and endothelial border.

In COVID-19 patients, all parts of the IIIS are strongly activated, especially in those who are admitted to the ICU with ARDS-like symptoms. Most morphological studies have been performed on lung tissue from autopsies of patients that had succumbed at the ICU, e.g. (20, 21). Macroscopic observations show that large sections of the pulmonary lobes were clogged with cells and fluid, explaining the poor oxygenation of the blood in severely ill patients. From these cases of seriously ill COVD-19 patients, it was observed that the pulmonary tissue was infiltrated with immune cells (20). Initially, lymphocytes and macrophages were reported to be the dominating cell types (22), but later studies also showed that PMNs were important players (23). This discrepancy was probably due to that the latter observation were obtained in patients with earlier disease. Another important finding is that the alveolar space is filled with plasma proteins, e.g., fibrinogen giving rise to fibrin clots and that the interalveolar capillaries are activated and obstructed with platelet-containing thrombi (**Figure 2**). A few cases with immunohistochemical studies of complement components have been performed (24, 25). They show deposition of C4d, C3d, factor B, sC5b-9 on the endothelium in various tissues, preferentially in the lung, which can be interpreted as if complement activation *via* the CP or/and the LP occurs with amplification *via* the AP. Taken together these findings are consistent with a widespread thromboinflammation in the lungs.

Late-stage severe COVID-19 primarily affects the lungs but extends to other organs causing, in the more serious cases, multiorgan impact including cardiac, central nervous system and kidney injury (26–28). Myocardial dysfunction, arrhythmia and acute coronary syndrome have been reported in association with COVID-19, but in a recently performed study on 30 patients with severe COVID-19 disease late cardiac pathology of biopsies showed no relevant cardiac histopathological alterations (29, 30). At present the contribution of SARS-CoV2 on cardiac lesions remains to be established (31). Activation of the complement system might be involved in the worsening of renal symptoms since deposition of C3 was significant in renal arteries and in glomerular capillaries of COVID-19 biopsies (32). This is supported by findings in our own study, which shows a correlation between C3a generation and glomerular filtration rate (1). Neurological disorders associated with COVID-19, such as outright encephalitis is rare but has been reported with direct SARS-CoV2 detection in brain tissue (33). However, severe COVID-19 with long ICU stay has been associated with critical illness involving neuro/myopathy (34) and post intensive care syndrome (35) where thromboinflammation may play a role.

## Thrombosis in COVID-19

Already early in the COVID-19 pandemic, it appeared that moderately to seriously ill patients showed a very strong involvement of the coagulation system, and a high incidence of thromboembolic complications was reported on both the arterial and the venous side. In order to prevent these complications, treatment of the patients with high doses (typically 150-300 IU/kg) of LMWH was used (13, 14). Initially it was thought that the cause of the thromboembolism was an effect directly targeting the coagulation system but there was no real evidence of a systemic activation. Morphological data from the lungs showed that defined sections of the tissue were rich in microcapillary thrombi suggesting rather that the clotting process was taking place locally close to the activated endothelium (18). The coagulation abnormalities that occur during COVID -19 disease were given a special name, COVID -19-associated coagulopathy (CAC), to emphasize what has emerged over time, that it is a condition with certain characteristics of its own. It is clear that CAC differs from the picture seen in sepsis and the so-called DIC (disseminated intravascular coagulation) where low platelet counts are a typical sign and extensive consumption of platelets and coagulation factors leads to thrombosis and bleeding at the same time (12, 36). Severely ill COVID -19 patients instead have normal or high platelet levels and are characterized by greatly elevated plasma levels of the fibrin degradation product D-dimer, which is a sensitive marker of fibrinolysis and coagulation turnover and probably reflects widespread thrombosis in e.g. small vessels in the lungs. It has been possible to reproduce the CAC phenotype in a primate model infected with SARS-CoV-2, and where an extensive fibrin deposition in the lung tissue was observed, in line with findings from autopsy materials of deceased COVID -19 patients (15, 37).

A number of hypotheses regarding the mechanisms behind CAC have been proposed. We have studied the relationship between complement and CAC, and shown that thrombosis in intensive care COVID -19 patients is linked to high activity in the LP and high plasma levels of the LP protein MBL (38). Future studies may show whether there is an underlying causal relationship between the LP and thrombosis, something that is supported by preclinical results where LP components MASP-1 and MASP-2 have a coagulation factor-like activity and can directly activate fibrinogen and prothrombin in *in vitro* experiments (39). There are also reports that neutrophil extracellular traps (NETs) that are released in response to C5a may be an initiator of coagulation since NETs expose TF and bind FXII (40). Similarly, activated endothelial cells express TF, which may contribute to the thromboinflammation.

## Activation Individual Components of IIIS in COVID-19

In COVID-19 an acute phase reaction increases the concentration of a number of plasma proteins multi-fold and thereby facilitates IIIS activation (41). Multiple proinflammatory cytokines and chemokines such as TNF, IL-1β, IL-6, and MCP-1 are produced in response to C3a and C5a generation that further amplifies the production of acute-phase proteins (41, 42). The IIIS activation is multifactorial: The virus itself can activate the IIIS directly *via* the CP and LP of the complement system and *via* the KK system (43–45). Another likely activation mechanism is tissue damage in the lungs with apoptotic and necrotic cells that initiates the overactivation of IIIS in its function to eliminate cells or cell debris (46).

### The Complement System

The complement system was early suspected to be involved in the pathophysiology of severe COVID-19 disease (26, 47–51) and has thereafter been thoroughly investigated and shown to be involved in the disease and correlated with the severity of the disease. The first indication was reported early in the pandemic where increased levels of sC5b-9 were detected and a bit later a more complete report was published; both demonstrating that increased levels of sC5b-9 were associated with severe disease (52, 53). Thereafter, several comprehensive studies have been published, which assess a number of different complement parameters. These studies show that individual complement components increase and decrease in concentration during the course of the disease without any clear patterns (1, 54). For instance, C3 and C4 and other components such as C1q and MBL, and complement function (CP and AP) are elevated in some patients and decreased in others, indicating that both overexpression and complement activation with consumption occur. Overexpressed and elevated levels of factor B tend to be associated with severe COVID-19. In addition to sC5b-9 generation, formation of other activation markers occurs: C4d, Bb, C3a, and C3d,g are generated and seem to be activated early in the disease. The early trigger of complement activation is still unclear; most likely several activators elicit this response. Activation has been reported to be mediated by the intact virus *via* intracellular complement activation (55) but also by virus proteins expressed on the surface of infected cells. The virus itself has been shown to bind MBL, Ficolin-2 and Collectin-11, *via* its S- and N-proteins, with subsequent LP-mediated C3b and C4b deposition (44, 45). Structural parts of the virus have also been shown to bind C1q and trigger complement activation (43). COVID-19 is strongly associated with ischemic cells, apoptosis, and cell death (15, 20, 40), initially occurring in the lung tissue. These conditions lead to binding of MBL, MASP-2 and C1q to the cells (see below), which may be the combined initiators of the accelerating complement activation that occurs particularly in severely ill patients. In our own study, the C1q concentration is low (consumed) in several patients at admission to the ICU approximately at day 10, possibly caused by damaged cells in the lungs (1).

The combined data indicate that the maximum activation occurs at approximately day 10 coinciding with when patients are most likely to be admitted to the ICU. C4 and C3 consumption combined with C4d, C3a and C3d,g generation supports complement activation *via* CP/LP activation. Daily monitoring of C3d,g has been reported to predict outcome in patients hospitalized with COVID-19 in combination with SARS-CoV-2 nucleocapsid antigen, RNA in blood, IL-6, and CRP (56).

### The KK System

In several early reviews and *in vitro studies*, the KK system was suggested to participate in the thromboinflammation of COVID-19 (43, 57–59), without presenting any evidence of KK system activation *in vivo*. No direct evidence of such activation was published until reports from us, and Busch et al. showed strong KK system activation in severely ill ICU patients (1, 60). The KK system activation was assessed by consumption of prekallikrein, HMWK and FXII, and by increased levels of kallikrein/C1-INH complexes (1). The activation of prekallikrein and HMWK on the admission to the ICU was shown to have prognostic significance for survival and the need for mechanical ventilation, but also for other systemic and specific organ parameters, such as, poor kidney function.

### The Fibrinolytic System

A consequence of the ongoing coagulation activation is generation of fibrin from fibrinogen. In the clot that is formed, plasmin is generated, which cleaves fibrin into fragments including D-dimer. D-dimer is an important clinical biomarker for fibrinolysis and indirectly for clot formation. Due to its general availability, this marker was one of the first biomarkers of IIIS reported to increase in COVID-19 and the levels were found to be associated with disease severity (61).

### Granulocytes (PMN)

The neutrophilic granulocytes, which are found in the lungs early in the course of the disease (23),have a profound effect on the outcome of COVID-19 (23). Several drivers of cell infiltration exist and the anaphylatoxins of the IIIS (C3a and C5a) as well as BK are likely to be important contributors. Chemotactic effects are mediated by the specific receptors C3aR (C3a), C5aR1 and 2 (C5a) and by BKR1 and 2 (BK). These receptors also trigger activation of the PMNs, which release proteases and other granular proteins, e.g., elastase, myeloperoxidase and protein-arginine deiminase type 4. One specific effect related to PMNs is the generation of NETs, which are large, extracellular, web-like structures consisting of cytosolic and granule proteins with a scaffold of decondensed chromatin in which the majority of the DNA originates from the nucleus (62). NETs protect against infection but have also been associated with a number of other immunoregulatory events such as binding antimicrobial peptides and priming immune cells (62). In addition to this, NETs promote thrombotic events, since they bind both TF (40) and FXIIa (63) thereby being able to trigger both the extrinsic pathway of coagulation as well as the contact system and consequently the intrinsic pathway of coagulation. NET formation has been suggested to be an important promotor of thrombosis in the lungs of COVID-19 patients where C5a has been proposed to elicit the release of these morphological structures (60, 62).

### Platelets and Endothelial Cells

Increased platelet activation and platelet-monocyte aggregates were observed in COVID-19 patients but not in patients presenting a mild syndrome (64). Platelet counts tend to increase in COVID-19 patients and not be consumed and decrease in number as in patients with thromboembolism. This observed difference, which speaks against that the thrombi found in the lungs in COVID-19 patients are the result of embolism with a major thrombus formed elsewhere such as in deep vein thrombosis (DVT). Instead, it has been observed that COVID-19 patients exhibit reduced procoagulant platelet responses (65), but platelets are found in the thrombi formed in the lung capillaries either as a result of activated endothelial cells or NETs formation (40). Endothelial cells are poorly infected by Cov-Sars-2 since these cells express a low number of ACE-2 molecules (66), but generation of both C5a and BK by the nearby pneumocytes can initiate expression of TF on both endothelial cells (67)and neutrophils (40, 68) thereby promoting local thrombus formation.

## Ischemic Injury

Hypoxia is anticipated to occur, particularly in the lungs during COVID-19, where major parts of the small airways may be totally clogged with fibrin, platelet-rich thrombi, and cells [**Figure 2**; (46)]. Ischemia is expected to be a mechanism that is involved in the injury mediated by the IIIS in COVID-19, considering the low oxygen saturation in these patients. Ischemia is a major stress to the cell, which can lead to changes in the composition and protein expression of the cell membrane (62). Consequently, IIIS in contact with a cell subjected to hypoxia, can recognize the cell surface as foreign, causing a thromboinflammatory reaction and, by extension, ischemia/reperfusion injury-like damage and cell death. The ischemic cell has a distinctive phenotype compared to the native one, which leads to that IIIS recognition molecules of the complement and the KK systems target the cell as foreign (non-self). In ischemia/reperfusion injury, MBL (69) and MASP-2 (70) of the LP and innate IgM antibodies (71, 72) of the CP have been suspected to be involved in the IIIS activation that occurs.

## The Renin-Angiotensin System (RAS)

Hypertension is linked to the renin-angiotensin system (RAS) and an increased risk for severe COVID-19 infection. The docking protein for SARS-CoV-2 on human cells is angiotensin converting enzyme (ACE)-2 of the angiotensinogen cascade system, which may destroy the function of this protein. ACE-1 inhibitors (common hypertensive drugs) block the cleavage of angiotensin I to angiotensin II. ACE-1 is a regulator of BK, making it feasible that inhibition of ACE-1 could aggravate the ARDS condition in COVID-19 patients by increasing the levels of active BK. Although in a meta study focusing on the effects of renin-angiotensin system (RAS) inhibitors on the RAS and the outcome of COVID-19, no support for this concept was found. However, since this study is based on several clinical trials that treat RAS inhibitors as a common group further studies are needed to elucidate this issue (58, 73).

## Conceptual Mechanisms Inducing COVID-19-Triggered ARDS

SARS-CoV-2-infected and ischemic, damaged epithelial and endothelial cells can activate all the IIIS cascade systems in a joint thromboinflammatory reaction in the lungs (**Figure 2**). During the development of ARDS, SARS CoV-2 and damaged cells (SARS CoV-2-infected, apoptotic, necrotic cells) are potential targets for the recognition molecules of the blood cascade systems. C1q, MBL, and FXII are known to recognize apoptotic and necrotic cells that can trigger the CP and LP of complement ultimately leading to cleavage of C3/C5 into C3a/C5a and C3b/C5b. BK generated by the KK system activation can cause dry cough (74) and pulmonary inflammation with edema (75) by binding to BKR2. C3a and C5a bind to the anaphylatoxin receptors: C3aR, C5aR1, and C5aR2 and C5a is also able to activate endothelial cells in COVID-19 patients leading to von Willebrand Factor (vWF) and p-selectin exposure on the endothelial lining thereby contributing to the thrombotic phenotype (76). Supporting the importance of the C5a/C5aR1 axis is that COVID-19 patients generate C5a as detected in pulmonary lavage in proportion to the severity of the disease and high expression of C5aR1 was found in blood and on pulmonary myeloid cells (77). C5a also induces expression of BKR1, (which contrary to BKR2 is not constitutively expressed) and BK can then act *via* both BKR2 (expressed on both the pulmonary epithelium and endothelium) and after cleavage to desArg9-BK *via* BKR1 (78–80), which also elicits increased vascular permeability, PMN chemotaxis, and nerve end stimulation, amplifying all of the above-mentioned reactions. Thus, C3a, C5a, and BK combined have the potential to cause a local edema and pulmonary leukocyte infiltration/inflammation that increases the distance between epithelial and endothelial cells in the alveolae, thereby hindering oxygenation of the blood (**Figure 2**). The poor oxygenation can lead to further ischemia (as described above) that in turn leads to even more IIIS activation thereby creating a vicious circle. Local thrombosis in the vasculature of the lung induced by activated endothelial cells that bind MBL, and expose selectins, TF and vWF on their surfaces, further aggravates the hypoxia. Chemotaxis and activation of neutrophlic granulocytes also cause NET formation and FXII activation (40, 81). Taken together these phenomena are likely drivers of the endothelialitis, the thrombus formation and the increased vascular angiogenesis linked to COVID-19 patients (15).

Activation of the IIIS in COVID-19-induced ARDS have many similarities to ARDS of other etiologies, e.g., as in sepsis but there are also large differences both regarding activation mechanisms and the focus of the inflammation. We have previously reported that the activation of the IIIS in COVID-19 mainly occurs *via* the KK system and the LP and CP of the complement system (1). By contrast, in septic shock, it has been reported by others that the key steps of complement activation consist of first the AP followed by the CP (36). One explanation of these discrepancies may be the specific pathophysiologic process in SARS-CoV-2 infection as compared with the diverse and heterogeneous microbiology in sepsis of different origin. Another important dissimilarity between COVID-19 and sepsis-induced ARDS is the focus and origin of inflammation. In COVID-19, ARDS originates from the lung, which is in contrast to sepsis-induced ARDS that is caused by a secondary systemic inflammatory response in a distant organ, unless the infection focus already is in the lung (82, 83). In the latter case, usually termed pulmonary ARDS, the lung is the driver of IIIS activation with the highest IIIS activation locally, while in the former, usually termed extrapulmonary ARDS, the lung is only secondarily affected and IIIS activation measured as activation products in the plasma is less dependent on the severity of ARDS.

In summary, we hypothesize that activation of the IIIS, either caused directly by SARS-CoV-2, or more likely by large quantities of activated and damaged cells resulting from viral infection and ischemia, is the pathophysiological mechanism of the thromboinflammatory reaction that triggers the ARDS linked to COVID-19.

## Therapeutics

A number of investigators have suggested the complement and KK systems as targets for treating COVID-19 (84). In addition to previously known treatment of IIIS activation *via* inhibition of the coagulation system (heparin, warfarin, direct-acting oral anticoagulants [DOAC], etc.), a large number of inhibitors have recently been developed to regulate the complement system. There are several licensed drugs that affect the components of the IIIS. Treatment of paroxysmal noctural hemoglobinuria (PNH), atypical hemolytic uremic syndrome (aHUS) and myasthenia gravis has been performed for a long time by inhibiting complement factor C5 (using the anti-C5 antibodies eculizumab and ravulizimab) with excellent results (85–87). Also, several other anti-complement drugs are being developed for use in, for example, treatment of COVID-19. Two of these AMY-101 and pegcatacoplan (both C3 inhibitors of the compstatin family) were recently tested and AMY-101 shown promising results with faster clinical recovery and a reduction in a number of inflammatory parameters such as plasma levels of IL-6 (88). In addition, the LP inhibitor narsoplimab (anti-MASP-2) has been tested in a small number of patients (89).

Inhibition of the KK system appears to be a possible step in the search for therapeutic alternatives for treatment of COVID-19. In support of this hypothesis are several publications including the successful treatment of ARDS with icatibant in hantavirus infection (90). The latter indicates that this may be a route of success, since it confirms that a BKR2 antagonist alleviated other types of virus-induced ARDS. Several currently registered drugs in clinical use for the treatment of angioedema target the KK system, which include icantibant, the kallikrein inhibitors lanadelumab (monoclonal antibody) and ecallantide (small molecular inhibitor), which is licensed in the USA. Also, C1-INH (both recombinant and in purified form), which inhibits both the complement and the KK systems, are examples of pharmaceuticals with a potential use to control IIIS activation in, e.g., COVID-19 patients. In a case control study C1-INH was investigated and shown to be safe and have some clinical effect (91). In a case study without controls (92) with recombinant C1-INH (conestat alfa) the drug was well taken but the study was indecisive. Considering reports of very high concentrations of C1-INH in COVID-19 patients, this finding may explain this result (60, 92).

In summary, all IIIS drugs were promising and had the expected effect on the targeted IIIS components and provided evidence that they affected thromboinflammation induced by IIIS to a varying degree. However, most of the studies included few patients without controls, which made evaluation of the results difficult. Further randomized and controlled studies will give us a deeper insight into the effect of these drugs alone. However, reflecting on the content of this review, combinations of IIIS inhibitors are most likely to be needed to get an optimal effect on COVID-19 ARDS. All trials currently registered in ClinicalTrials.gov testing IIIS-targeted components in COVID-19 are summarized in **Table 1**.

**Table 1:** All trials currently (2022-01-28) registered in ClinicalTrials.gov testing targeting IIIS components in COVID-19.

Drug (target)	Identifier	Participants	Study design	Last update
**Kallikrein**				
lanadelumab (kallikrein)	NCT04422509	43	Randomized vs SOC	Nov 16, 2021
lanadelumab (kallikrein)	NCT04460105	0	Randomized vs placebo	Oct 20, 2020
ISIS 721744 (kallikrein antisense)	NCT04549922	111	Randomized vs placebo	April 19, 2021
**Icatibant**				
C1-INH ± icatibant	NCT05010876	44	Randomized vs SOC	Aug 18, 2021
Iactibant	NCT04978051	120	Randomized vs SOC	July 27, 2021
**C1-INH**				
Conestat alfa (recomb C1-INH)	NCT04414631	80	Randomized vs SOC	Nov 9, 2021
Ruconest (recomb C1-INH)	NCT04705831	40	Randomized vs SOC, crossover	Jan 12, 2021
Ruconest (recomb C1-INH)	NCT04530136	120	Randomized vs SOC	Dec 10, 2020
**C5 cleavage inhibitors**				
Eculizumab	NCT04346797	120	Randomized vs SOC	April 20, 2020
Eculizumab	NCT04288713	no info	no info found	March 20, 2020
Ravulizumab	NCT04390464	1167 (3 arms)	Randomized vs SOC	May 18, 2020
Ravulizumab	NCT04570397	32	Randomized vs SOC	Jan 14, 2021
Ravulizumab	NCT04369469	120	Randomized vs SOC	Sept 22, 2021
Zilucoplan (C5 cleavage inhibiting peptide)	NCT04382755	81	Randomized vs SOC + antibiotics	July 2, 2021
Zilucoplan (C5 cleavage inhibiting peptide)	NCT04590586	516 (7 arms)	Randomized vs SOC + placebo	Nov 23, 2021
**C3 cleavage inhibitors**				
AMY-101	NCT04395456	144	Randomized vs placebo	Feb 20, 2021
APL-9	NCT04402060	65	Randomized vs placebo	Sept 1, 2021
Lectin pathway inhibitor				
Narsoplimab (anti MASP-2)	NCT04488081	1500 (8 arms)	Randomized	July 21, 2021
+ Remdesivir (anti CD14)				
**C5aR antagonists**				
avdoralimab (anti C5aR mAb)	NCT04371367	208	Randomized vs placebo	May 27, 2021
avdoralimab (anti C5aR mAb)	NCT04333914	219	Randomized vs SOC	Aug 5, 2021
vilobelimab (anti C5a mAb)	NCT04333420	390	Randomized vs SOC + placebo	Dec 31, 2021

SOC, standard of care.

## Author Contributions

BN and KE have written and edited the major part of the review. OE, KF, and MH-L have contributed to the writing and editing of the article. All authors contributed to the article and approved the submitted version.

## Funding

The study was funded by the Swedish Research Council grants 2016-01060, 2016-04519, 2020-05762, 2021-02252, the Swedish Heart-Lung Foundation grant HLF 2020-0398 and by faculty grants from the Linnaeus University.

## Conflict of Interest

The authors declare that the research was conducted in the absence of any commercial or financial relationships that could be construed as a potential conflict of interest.

## Publisher’s Note

All claims expressed in this article are solely those of the authors and do not necessarily represent those of their affiliated organizations, or those of the publisher, the editors and the reviewers. Any product that may be evaluated in this article, or claim that may be made by its manufacturer, is not guaranteed or endorsed by the publisher.

## Abbreviations

ACE, *angiotensin converting enzyme;* aHUS, atypical hemolytic uremic syndrome; AP, alternative pathway of complement; ARDS, acute respiratory distress syndrome; BK, bradykinin; BKR1/2, bradykinin receptor 1/2; C1-INH, C1 inhibitor; CAC, COVID-19-associated coagulopathy; CP, classical pathway of complement; CT, computed tomography; DAMPs, damage-associated molecular patterns; DIC, disseminated intravascular coagulation; DOAC, direct-acting oral anticoagulants; DVT, deep vein thrombosis; FXII, Factor XII; ICU, intensive care unit; HMWK, high molecular weight kininogen; IIIS, intravascular innate immune system; KK, kallikrein/kinin system; LMWH, low-molecular-weight heparin; LP, lectin pathway of complement; MERS, middle East respiratory syndrome; NETs, neutrophil extracellular traps; PAMPs, pathogen-associated molecular patterns; PMNs, polymorphonuclear leukocytes; PNH, paroxysmal noctural hemoglobinuria; RAS, renin-angiotensin system; SARS-1, severe acute respiratory ryndrome-1; TF, tissue factor; tPA, tissue plasminogen activator; vWF, von Willebrand factor.
